# The Temporal Dynamics of Perceiving Other’s Painful Actions

**DOI:** 10.3389/fpsyg.2016.01847

**Published:** 2016-11-22

**Authors:** Fang Cui, Ruolei Gu, Xiangru Zhu, Yue-jia Luo

**Affiliations:** ^1^Institute of Affective and Social Neuroscience, Shenzhen University, ShenzhenChina; ^2^CAS Key Laboratory of Behavioral Science, Institute of Psychology, BeijingChina; ^3^Institute of Cognition and Behavior, Henan University, KaifengChina; ^4^Department of Psychology, Southern Medical University, GuangzhouChina; ^5^Shenzhen Institute of Neuroscience, ShenzhenChina

**Keywords:** event-related potentials (ERPs), action perception, pain, sensory expectation

## Abstract

The present study investigates the temporal dynamics of the brain activity predicting the sensory outcomes of observed hand–object interactions of others. Participants are presented with pictures of a hand grasping or withdrawing from noxious and neutral objects. They are then asked to judge whether this hand–object interaction causes painful consequences. In the early stages of stimulus processing, the effect of action was observed in the event-related potential components N1 and N2. Significant interactions of action × object were observed in the later components P3 and late positive potential (LPP): only when the object was noxious, the action “grasp” elicited a significantly larger amplitude than the action “withdrawal”. These results suggest that: on the one hand, when observing the hand–object interaction from the third-person perspective, the action type of others can be processed in an automatic style. On the other hand, integrating the information of action and object to predict the sensory consequence of this interaction is a top–down, cognitive controlled processing. The current findings are different from previous studies using first-person perspective visual stimuli which support that the processing of hand–object interaction is rapid and automatic.

## Introduction

In our daily life, we constantly witness others handling objects. As social animals, we can derive the information about objects, contexts, and even mental and emotional states of other people from observing these hand–object interactions and predict the sensations that are associated with these actions (Morrison et al., 2013). For example, when we saw a person reaching his hand to grasp the sharp end of a knife, we know he would feel pain before we actually saw his hand touched the knife.

Knowing others in pain requires the ability of empathy. Neuroimaging evidence suggests that there are two components of empathy subserved by distinct brain networks (Decety and Lamm, 2006). Specifically, the affective component of empathy has been framed as reflecting rapid bottom–up activations of the limbic system (Keysers et al., 2010, 2014; Rizzolatti and Sinigaglia, 2010; Lamm et al., 2011). The cognitive component of empathy, on the other hand, has been shown to be influenced by higher-level, top–down, signals originating in prefrontal cortical circuitries (Decety and Lamm, 2006; Keysers et al., 2010). Previous event-related potential (ERP) studies support this two-component model by revealing that the empathy for pain involves two key processes: an early, automatic (bottom–up) process related to perception–action coupling and a later, cognitive controlled (top–down) process (Fan and Han, 2008). ERP research has found that both early (N1 and N2) and later [P3/late positive potential (LPP)] ERP components are sensitive to the comparison of observing others receiving painful stimuli to non-painful stimuli (Fan and Han, 2008; Han et al., 2008, 2009; Meng et al., 2013).

Most visual stimuli used in studies of empathy for pain were static pictures showing the painful and non-painful consequences of other’s actions (Fan and Han, 2008; Han et al., 2008, 2009; Meng et al., 2013). It was found that painful and non-painful stimuli can be distinguished on the early ERP component N1 (peaked at ∼110 ms; Fan and Han, 2008). These studies suggested that the human brain can rapid and automatically discriminate whether another person was in pain or not. Some other studies using video-clips showing the dynamic process of a needle stabbing another person’s hand also found that the observation of the video automatically induces the covert simulation in the onlooker’s corticospinal system. These findings also support that the encoding of other’s pain is an automatic but not a top–down controlled process (Avenanti et al., 2005, 2009).

Regarding that, the next question is: how does the human brain make this prediction? Based on the “sensory expectation” theory, observing other’s hand–object interactions may involve both action representations and an “expectation” of how the object’s properties would affect the sensory surface of the acting person’s hand (Eickhoff et al., 2006; Dijkerman and de Haan, 2007; Colder, 2015). The processing of this hand–object interaction involves three aspects. First, different action types need to be differentiated. Second, the sensory-tactile qualities of the object need to be coded (e.g., whether the object is noxious or neutral). Third, the two aforementioned aspects of information need to be integrated in a predictive manner to represent the sensory outcome of this hand–object interaction (Eickhoff et al., 2006; Gazzola and Keysers, 2009). A recent functional magnetic resonance imaging (fMRI) study has provided neural evidence of the existence of these three aspects. In that study, participants observed other’s hands grasping or withdrawing from either noxious or neutral objects, and the results showed that distinct sensorimotor subregions represented preferential responses to different aspects of the stimuli: object noxiousness (noxious vs. neutral), action type (grasp vs. withdrawal), and painful action outcomes (painful grasps vs. all other conditions). More specifically, separate somatosensory/inferior partial lobule (IPL) subregions responded more strongly when the observed action targeted at a noxious object compared with a neutral object, regardless of the action type. Other subregions responded more strongly to observed grasps than to observed withdrawals, regardless of whether the object was noxious or not. Finally, a region in the somatosensory cortices was found to be activated only in the condition in which the hand–object interaction would cause a painful consequence (Morrison et al., 2013).

To our knowledge, the temporal aspects of this processing have not been explored yet. In the present study, we aimed to explore the temporal dynamics of this processing. We also noticed that all the visual stimuli (pictures or video-clips) used in previous experiments only showed views from the first-person perspective. Based on our daily experience, we know that human beings can also predict other’s pain precisely when the visual input was from the third-person perspective. Therefore in the current study, we also want to explore how the brain predicts other’s pain from a third-person perspective. The participants were presented with pictures that showed a hand grasping or withdrawing from a noxious or neutral object and were asked to judge whether the observed hand–object interaction could cause a painful consequence. We then compared ERPs when the participants observed different kinds of pictures.

The present study is exploratory, and previous literature on the same topic is limited. In our opinion, when observing pictures showing an ongoing hand–object interaction from the third-person perspective, the observers would not be able to distinguish painful consequence from non-painful consequence as quickly as in the first-person perspective condition. This is because the third-person perspective may prevent the participants from directly “embedded” other’s emotional feelings. Instead, they may need to encode the information of action types and object properties first, and then to integrate different information to finish the prediction. Therefore, we expected to find the main effect of action and/or object in the earlier stage of stimulus processing (such as the N1, P1, and N2 components), and find the interaction of action × object in the later stage (such as the P3 and LPP components). Meanwhile, whether the prediction of other’s pain is conducted in an automatic or a top–down cognitive-controlled style would depend on at which temporal stage we can find the significant interaction of action × object. Specifically, if the interaction of action × object was found on later ERP components (e.g., P3 and/or LPP), these results would suggest that the integration is a top–down process. Otherwise, it is possible that the integration process happened automatically.

## Materials and Methods

### Participants

Eighteen right-handed participants (10 male; 21.25 ± 0.73 years [mean ±*SE*]) with no history of neurological disorders, brain injury, or developmental disabilities participated in the study. All of them have normal or corrected-to-normal vision. The study was approved by the Medical Ethical Committee of Shenzhen University Medical School. All of the participants provided written informed consent. They received monetary compensation for participation.

### Stimuli

The stimuli consisted of 32 color pictures that showed a human hand grasping or withdrawing from a noxious or a neutral object. All of the objects appeared in the pictures can be grasped with a power grip. Two categories of objects were presented (noxious and neutral), with eight objects in each category (**Supplementary Table S1**).

**FIGURE 1:**
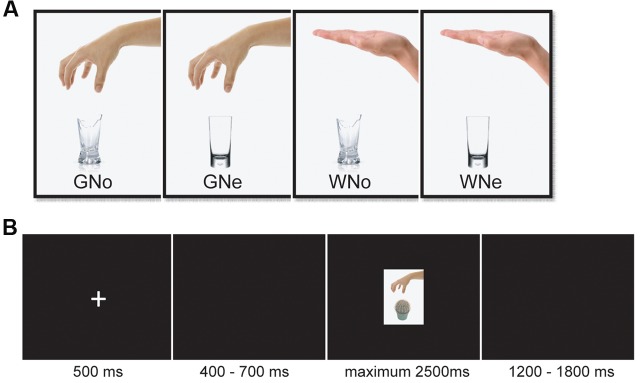
**(A)** Examples of pictures in each condition **(B)** an example from one trial.

The neutral and noxious objects in the pictures have been used in previous studies. The level of dangerousness of the objects has been evaluated in the original studies and the categorization of the noxious and neutral objects have been demonstrated to be valid (Anelli et al., 2012, 2013). There were four kinds of pictures: grasping a noxious object, grasping a neutral object, withdrawing from a noxious object, and withdrawing from a neutral object. Each kind consisted of eight different pictures (**Figure 1A** shows an example of each category). All of the pictures were identical with regard to size, background, contrast, brightness, and other physical properties. All of them were presented on a black background (3.0° × 3.5° of visual angle).

### Experimental Procedures

The stimulus display and behavioral data acquisition were performed using E-Prime 2.0 software (Psychology Software Tools). During the task, the participants sat comfortably in an electrically shielded room approximately 90 cm from a 15-inch color monitor. The participants were informed that they need to do a “predict pain” task. They would be presented with pictures showing a human hand grasping or withdrawing from an object. They had a maximum of 2.5 s to judge whether this hand–object interaction would cause a painful sensation in the executor. Each trial began with a fixation cross presented in the center of the screen for 500 ms followed by 400–700 ms interval. Then the picture appeared for a maximum of 2500 ms and disappeared until a response was given. If the participant believed the hand–object interaction shown in the picture would cause pain, then they press the “F” on the keyboard; otherwise, they press the “J”. The keyboard letter assignments were counterbalanced. There was a 1200–1800 ms inter-trial interval (**Figure 1B**). A total of 224 trials were conducted, evenly separated into two blocks. Each of the 32 pictures was repeated seven times.

The study utilized a 2 × 2 within-subjects design. The first factor was the object (noxious or neutral). The second factor was the action (grasp or withdrawal). The experiment had a total of four conditions: Grasp-Noxious (GNo), Grasp-Neutral (GNe), Withdrawal-Noxious (WNo), and Withdrawal-Neutral (WNe). After the participants completed the task, they were asked to rate the degree of painfulness for each picture on a 7-point Likert scale (1 = not painful at all and 7 = extremely painful).

### Electroencephalographic (EEG) Acquisition and Analysis

Electroencephalographic data were recorded from a 64-electrode scalp cap using the 10–20 system (Brain Products, Munich, Germany) with reference electrodes on the left and right mastoids. The vertical electrooculogram (EOG) was recorded with placed above and below the left eye. EEG and EOG activity was amplified at 0.01–100 Hz band-pass and sampled at 500 Hz. All of the electrode impedances were maintained below 5 kΩ.

The EEG data were pre-processed and analyzed using Matlab R2011b software (MathWorks) and the EEGLAB toolbox (Delorme and Makeig, 2004). The EEG data for each electrode were down-sampled to 250 Hz and re-referenced to the grand averages. The signal was then passed through a 0.01- to 30-Hz band-pass filter. Time windows of 200 ms before and 700 ms after the onset of the picture were segmented. EOG artifacts were corrected using an independent component analysis (ICA) (Jung et al., 2001) (**Supplementary Figure S1**). Epochs with amplitudes that exceeded ±50 μV at any electrode were excluded from the average (5.6 ± 0.6% trials were excluded).

### Data Measurement and Analysis

We calculated the accuracy (ACC) and reaction times (RTs) as the behavioral indices of the participants in this “predict pain” task. A within-subjects repeated-measures analysis of variance (ANOVA) was performed, with action and object as two within-subjects factors. The participants were asked to rate “How painful would the hand–object interaction be?” for all pictures. The ratings for the four conditions were analyzed using the same within-subject repeated-measures ANOVA. All of the analyses were performed using SPSS 22 software.

The follow-up analyses focused on the ERPs elicited by observing pictures. The averaged epoch was 900 ms, including a 200 ms pre-stimulus baseline. Since the current study is exploratory, the statistical analysis was conducted at electrodes selected from five regions that covered the whole scalp: frontal (Fz, FCz, F3–F4, and FC3–FC4), central (Cz, CPz, C3–C4, and CP3–CP4), parietal (Pz and P3–P4), temporal (T7–T8, TP7–TP8, and P7–P8), and occipito-temporal (POz, Oz, PO3–PO4, and PO7–PO8) regions (Fan and Han, 2008). Based on the literature, observing affective picture can trigger ERP components from short (N1, P2, and N2) to long (P3 and LPP) latencies (for a review: Schupp et al., 2000). In the current dataset, the mean ERP waves from each regions and the topographical distributions were inspected to determine the characteristics of aforementioned components, specifically, the N1 (90–140 ms), P2 (160–200ms), N2 (240–300 ms), P3 (250–350 ms), and the LPP (400–550 ms) components. The peak amplitudes for each time window from all of the five regions were subjected to a three-way repeated-measures ANOVA with object (noxious and neutral), action (grasp and withdrawal), and region (frontal, central, parietal, temporal, and occipital–temporal) as within-subjects factors. The degrees of freedom for *F*-ratios were corrected according to the Greenhouse–Geisser method. Differences were considered statistically significant at *p* < 0.05. For the sake of brevity, only significant effects are reported hereinafter.

## Results

### Behavioral Data

For the ACC, we found a significant main effect of action (*F*_1,17_ = 33.551, *p* < 0.001, and $\eta_{p}^{2}$ = 0.664); when the action was “withdrawal”, the ACC of judgment was significantly higher than when the action was “grasp”(grasp: 93.2 ± 0.7% and withdrawal: 97.3 ± 0.6%). We also found a significant main effect of object (*F*_1,17_ = 5.369, *p* = 0.033, and $\eta_{p}^{2}$ = 0.240); when the object was neutral, the ACC was significantly higher than when the object was noxious (neutral: 96.5 ± 0.6% and noxious: 94.1 ± 0.9%). The interaction of action × object was not significance (*F*_1,17_ = 0.235, *p* = 0.634, and $\eta_{p}^{2}$ = 0.014).

For the RT, a significant main effect of action (*F*_1,17_ = 35.953, *p* < 0.001, and $\eta_{p}^{2}$ = 0.679) was observed: when the action was “withdrawal”, RT was significantly shorter than when the action was “grasp” (grasp: 778.690 ± 27.873 ms; withdrawal: 669.258 ± 29.518 ms). A significant main effect of object (*F*_1,17_ = 5.041, *p* = 0.038, and $\eta_{p}^{2}$ = 0.229) was also found to be significant: when the object was neutral, the RT was significantly shorter than when the object was noxious (neutral: 708.237 ± 28.771 ms and noxious: 739.711 ± 27.426 ms). The interaction of action × object was close to significant (*F*_1,17_ = 3.754, *p* = 0.069, and $\eta_{p}^{2}$ = 0.181).

For the subjective rating of painfulness of the hand–object interaction, we found a significant main effect of action (*F*_1,17_ = 235.003, *p* < 0.001, and $\eta_{p}^{2}$ = 0.933); when the action was withdrawal, the rating was significantly lower than when the action was grasp (grasp: 3.855 ± 0.123 and withdrawal: 1.306 ± 0.084) as well as a significant main effect of object (*F*_1,17_ = 289.346, *p* < 0.001, and $\eta_{p}^{2}$ = 0.945); when the object was neutral, the rating was significantly lower than when the object was noxious (neutral: 1.378 ± 0.066 and noxious: 3.783 ± 0.118).

### ERP Data

*N1*. The main effect of region was significant (*F*_4,68_ = 7.788, *p* = 0.003, and $\eta_{p}^{2}$ = 0.314) indicating that N1 reached its maximum in the frontal region (frontal: -2.533 ± 0.266 μV, central: -1.481 ± 0.190 μV, parietal: -0.475 ± 0.398 μV, temporal: -0.363 ± 0.271 μV, and occipito-temporal: -0.067 ± 0.604 μV). The main effect of action was significant (*F*_1,17_ = 15.566, *p* = 0.001, and $\eta_{p}^{2}$ = 0.478): the amplitude was more negative when the action was “grasp” than when the action was “withdrawal” (grasp: -1.156 ± 0.202 μV and withdrawal: -0.811 ± 0.181 μV).

**FIGURE 2:**
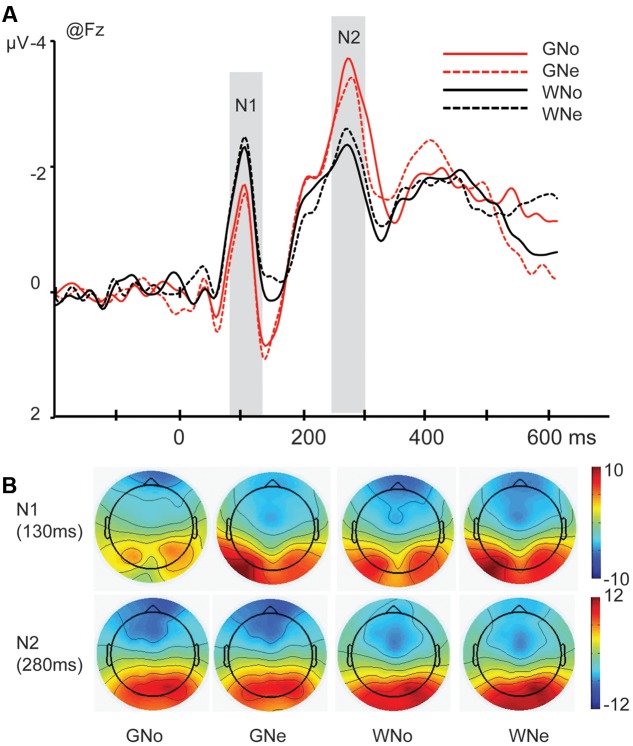
**(A)** Grand average of N1 and N2 components at Fz site in the four conditions; **(B)** voltage scalp maps for N1 and N2 in four conditions

The interaction of action × region was significant (*F*_4,68_ = 10.028, *p* < 0.001, and $\eta_{p}^{2}$ = 0.371). Pairwise comparisons revealed that in the frontal region, where the N1 reached its maximum, withdrawal elicited a significantly larger negativity than grasp (grasp: -2.249 ± 0.277 μV; -withdrawal: -2.818 ± 0.277 μV, *p* = 0.002; **Figures 2A,B**).

*P2*. A significant main effect of region was observed (*F*_4,68_ = 7.986, *p* = 0.006, and $\eta_{p}^{2}$ = 0.320), indicating the P2 reached its maximum in the occipito-temporal region (frontal: 0.911 ± 0.543 μV, central: -0.177 ± 0.230 μV, parietal: 1.381 ± 0.457 μV, temporal: 1.042 ± 0.365 μV, and occipito-temporal: 3.957 ± 0.835 μV).

The interaction of action × region was significant (*F*_4,68_ = 6.697, *p* = 0.001, and $\eta_{p}^{2}$ = 0.283). Pairwise comparisons showed that in the occipito-temporal region where the P2 reached its peak, the difference between two actions were insignificant (grasp: 3.898 ± 0.803 μV; withdrawal: 4.257 ± 0.888 μV, *p* = 0.077). Therefore, we cannot conclude the effect of action was significant in P2.

*N2*. The main effect of region was significant (*F*_4,68_ = 17.027, *p* < 0.001, and $\eta_{p}^{2}$ = 0.500), indicating that the N2 mainly distributed in the frontal region (frontal: -2.522 ± 0.342 μV, central: -0.056 ± 0.322 μV, parietal: 1.911 ± 0.376 μV, temporal: -0.218 ± 0.369 μV, and occipito-temporal: 2.004 ± 0.667 μV).

The interaction of action × region was significant (*F*_4,68_ = 4.487, *p* = 0.027, and $\eta_{p}^{2}$ = 0.209) and pairwise comparisons revealed that in the frontal region where the N2 reached its peak, grasp elicited a significantly larger negativity than withdrawal (grasp: -2.817 ± 0.293 μV; withdrawal: -2.228 ± 0.417 μV, *p* = 0.018; **Figures 2A,B**).

*P3*. A significant main effect of region was observed (*F*_4,68_ = 26.788, *p* < 0.001, and $\eta_{p}^{2}$ = 0.612), indicating that the amplitude of P3 component was maximum in the parietal region (frontal: -0.530 ± 0.366 μV, central: 2.302 ± 0.274 μV, parietal: 4.972 ± 0.358 μV, temporal: 1.249 ± 0.330 μV, and occipito-temporal: 2.341 ± 0.661 μV).

**FIGURE 3:**
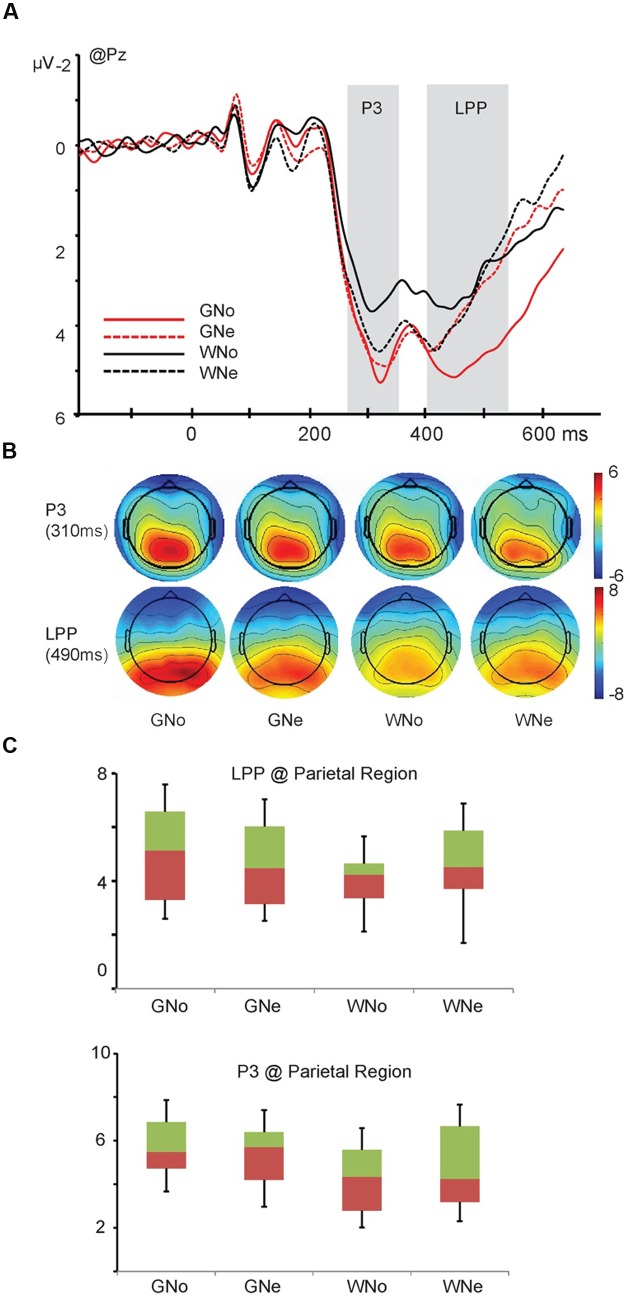
**(A)** Grand average and voltage scalp map of P3 and late positive potential (LPP) component at Pz; **(B)** voltage scalp map of P3 and LPP components **(C)** the interaction of action × object of P3 and LPP components in the parietal region.

The interaction of action × region was significant (F_4,68_ = 9.121, *p* < 0.001, and$\eta_{p}^{2}$ = 0.349). Pairwise comparisons indicated that in the parietal region, grasp elicited a significantly larger negativity than withdrawal (grasp: 5.476 ± 0.374 μV; withdrawal: 4.469 ± 0.401 μV, *p* = 0.004). A three way interaction of action × object × region was significant (*F*_4,68_ = 3.518, *p* = 0.033, and $\eta_{p}^{2}$ = 0.171). Pairwise comparisons revealed that in the parietal region, only when the object was noxious, the action “grasp” elicited a significantly larger negativity than the action “withdrawal” (grasp: 5.529 ± 0.407 μV; withdrawal: 4.176 ± 0.391 μV, *p* = 0.001); when the object was neutral, the difference between two action types was insignificant (grasp: 5.422 ± 0.365 μV; withdrawal: 4.762 ± 0.439 μV, *p* = 0.074; **Figures 3A–C**).

*LPP*. The main effect of region was significant (*F*_4,68_ = 21.910, *p* < 0.001, and $\eta_{p}^{2}$ = 0.563), indicating the amplitude of the LPP was maximum in the parietal region (frontal: -0.199 ± 0.349 μV, central: 2.759 ± 0.250 μV, parietal: 4.609 ± 0.413 μV, temporal: 1.294 ± 0.341 μV, and occipito-temporal: 3.545 ± 0.621 μV). The main effect of action was significant (*F*_1,17_ = 5.290, *p* = 0.034, and $\eta_{p}^{2}$ = 0.237): the amplitude was larger when the action was “grasp” than when the action was “withdrawal” (grasp: 2.487 ± 0.205 μV and withdrawal: 2.316 ± 0.206 μV).

A three way interaction of action × object × region was significant (*F*_4,68_ = 5.096, *p* = 0.008, and $\eta_{p}^{2}$ = 0.231). Pairwise comparisons revealed that in the parietal region, only when the object was noxious, the action “grasp” elicited a significantly larger LPP amplitude than the action “withdrawal” (grasp: 5.188 ± 0.447 μV; withdrawal: 4.029 ± 0.404 μV, *p* < 0.001); when the object was neutral, the difference was insignificant (grasp: 4.618 ± 0.390 μV; withdrawal: 4.600 ± 0.517 μV, *p* = 0.953; **Figures 3A–C**).

## Discussion

The present study aimed to explore the temporal dynamics of predicting the expected sensory consequences of the observed hand–object interactions of others. Participants were asked to watch others’ hands either grasping or withdrawing from objects that were either noxious or neutral and judged whether this interaction would cause painful consequences. The application of the ERPs allowed us to explore in which temporal stage of stimulus processing, the object (noxious or neutral), action (grasp or withdrawal), and their integration (whether this hand–object interaction would cause pain) were evaluated.

Regarding the behavioral results, it was suggesting that when the action type or the property of object in the hand–object interaction has the potential to cause harm, the prediction task became more difficult for the participants. It is worth noticing that we do not observe any significant action × object interaction in behavioral data. Though we do found the interaction of action × object was close to significance in the RTs (*p* = 0.069). We can tell from the data that when the action was withdrawal, the difference between neutral and noxious object was relatively larger than when the action was grasp (**Table 1**). The insignificance may due to the limited sensitivity of behavioral measurements.

**Table 1:** The first four columns present the two factors in the design and coding of each condition.

	Action	
	
	Grasp	Withdrawal
**ACCs (%)**		
Neutral object	93.73 ± 5.10	99.19 ± 1.76
Noxious object	92.74 ± 4.70	95.50 ± 4.14
**RTs (ms)**		
Neutral object	783.64 ± 126.62	632.93 ± 126.02
Noxious object	773.74 ± 125.16	705.68 ± 131.55
**Ratings**		
Neutral object	1.713 ± 0.56	1.042 ± 0.11
Noxious object	5.996 ± 0.73	1.571 ± 0.67


*The latter three columns present accuracy (ACC) and reaction times (RTs) for each condition. The last column is the subjective rating of the degree of painfulness for each kind of picture stimulus (mean ±*SD*)*

With regards to the ERP data, on the N1 component (peaked at 110 ms) we found that the action “withdrawal” elicited significantly larger negative amplitude than the action “grasp”. This effect indicates that object-oriented actions (grasp) and non-object-oriented actions (withdrawal) are differentiated in the early visual processing stage. The N1 component may reflect the activity of a neural population that is involved in the early integration of agent form (e.g, a human being or a robot) and motion type (Baccus et al., 2009). Previous studies have found that neural responses to the onset of movements of the mouth and eyes could be observed within 200 ms after motion onset (Puce et al., 2000). Similar results were found for the observation of whole-body actions (e.g., walking) of others (Wheaton et al., 2001). This effect may suggest that the human visual system is very efficient in detecting human actions in a visual scene (Jokisch et al., 2005).

The effect of action was also observed on the component N2 (peaked 280 ms) where the “grasp” elicited significantly larger amplitudes than the “withdrawal”. The literature suggests that high arousing (regardless of its emotional valence) stimuli elicit a more pronounced N2 than low arousing (neutral) stimuli (Olofsson and Polich, 2007), which may indexes an evolutionarily adaptive attentional bias such that the evaluation of image features is inclined to affectively arousing stimuli for further processing (Schupp et al., 2000; Dolcos and Cabeza, 2002). In the current study, “grasp” would be more arousing than “withdrawal” because the former one is an object-oriented action which needs further processing.

An important human ability is responding properly to various objects in the environment. Previous behavioral and psychophysiological studies suggest that people are sensitive to the differences between noxious and neutral objects (Anelli et al., 2012, 2013). In the present data, we do not observe any effect of object in the early components such as N1, P2, and N2, implying that the early stages of stimulus processing are dominated by the processing of action but not the processing of object. These findings are consistent with a previous study which found significantly larger P3 and LPP when phobic participants were presented with fearful stimuli compared with control participants but no effect in the early ERP components (Miltner et al., 2005). The literature suggests that objects in the environment are taken into consideration when they potentially offer an opportunity to a subject or might signal a threat, in either case the salience of an object depends upon the motivation of the subjects. The key of human instinct is not to respond to objects per se, but to evaluate alternative actions in a given context (Mirabella, 2014). Regarding that, the human brain is more likely to process an object when it is the target of somebody. Although no effect was observed in the ERP result to indicate the encoding of object property alone, we should note that being aware of the potentially dangerous object in the circumstance is crucial for surviving. Therefore, when they appear in the sight, they can distract attention from the ongoing task. Regarding the behavioral results, when the object was noxious, the task was performed more slowly and with lower ACC comparing to when the object was neutral. The subjective evaluation also reflect the harmfulness of the object, that is, even when the other’s hand was withdrawing from the object, the observer still felt danger.

Finally, to readily predict the sensory consequence of others’ actions, observers not only need to encode the action type or/and the object property but also need to integrate these two aspects of information to form a prediction. Neuroimaging evidence suggested that different brain regions underlie the encoding of action, the encoding of object, and the integration of the two aspects in the judgments of an action’s appropriateness (e.g., whether this hand–object interaction would cause a painful consequence) (Morrison et al., 2013). From the temporal aspect, the current results find significant interactions between action and object in the later components P3 and LPP. For both the P3 and LPP, only when the object was noxious, the action “grasp” elicited significantly larger amplitudes than the action “withdrawal”; but when the object was neutral, the difference between these two types of action was insignificant. These results indicate that in the temporal dimension, the interaction of action and object happens later than the encoding of action and it is a cognitive-controlled process. However, as we know that pain is intimately linked with action systems, so as to allow people to freeze or escape for survival. Regarding that, if pain (and empathy for pain) guides adaptive homeostatic responses, the processing of painful stimuli must be very quick (LeDoux, 2014). Previous studies also suggest that empathy for pain is an automatic but not a top–down process (Avenanti et al., 2005, 2009). Then why in the present study we find that predicting other’s pain is not automatic? We propose that this question could be explained by the perspective of the presentation of the stimuli. The visual stimuli used in previous studies all show views from the first-person perspective while in the current study the pictures show view from the third-person perspective. Compared to the first-person perspective, when viewing the scene from the third-person perspective, it may be more difficult for the observers to directly put themselves into other’s shoes. Consistent with this point of view, a recent behavioral study finds that participants detected a tactile object that was delivered to other’s hand when the stimuli were presented from a first-person perspective, but not when the stimuli were presented from a third-person perspective. To explain these findings, the authors suggest that to empathize with other’s feelings, the sensory consequences of the other’s actions need to be represented in the observer’s own tactile representation system; in the third-person perspective these effects are restricted by basic components of the others’ actions, such as the object properties and action types. More high-level components, such as the integration of the object and action for predicting sensory consequences cannot be processed directly but need to be transferred to the first-person perspective first(Bach et al., 2014). Accordingly, we suggest that the current study offers psychophysical evidence for this assumption and proves that when the stimuli are presented from the third-person perspective, the integration of the action and object may involve more top–down processing.

In summary, the present study has found that when predicting the sensory consequence of other’s hand–object interaction, the action type is encoded in the early stages of stimulus processing (manifested on the N1 and N2 components). Subsequently, the interaction of action type and object property happens mainly in the later stage of stimulus processing (manifested on the P3 and LPP). The current results also suggest that when observing an ongoing hand–object interaction from the third-person perspective, the prediction of other’s pain is more likely to be a cognitive controlled top–down process, rather than an automatic one.

### Limitations

There are two limitations of the present study which should be noted. First of all, we used static pictures to present a dynamic action. Before the task, we informed the participants that they would observe pictures showing an ongoing action. In addition, the feedback questionnaire has proved that the participants did feel they were observing ongoing hand actions. However, there are still obvious difference between static pictures and movies that showing dynamic actions. Future studies should consider using active stimuli to explore this topic. Second, in all the stimuli used in the current study, all the hands were placed in the top half of the picture and objects were placed in the bottom half. Consequently, the participants might have paid attention to the hand first. In future studies, we should balance the location of hand and object to avoid this potential confound.

## Author Contributions

FC contributed in designing the experiment, analyzing the data, and writing the manuscript. RG contributed in analyzing the data and writing the manuscript. XZ contributed in collecting the data and analyzing the data, and YL contributed in writing the manuscript.

## Conflict of Interest Statement

The authors declare that the research was conducted in the absence of any commercial or financial relationships that could be construed as a potential conflict of interest.

## Supplementary Material

**FIGURE S1 | (A)** The EEG signal before and after using independent component analysis (ICA) removing the artifact of eye blink and **(B)** the component reflecting the eye blinks identified using ICA (marked with yellow circle).

**TABLE S1 | Two categories of objects: noxious and neutral with eight objects in each category.**
