# Supplementary figures and images for: The Temporal Dynamics of Perceiving Other’s Painful Actions
Source: Front Psychol. 2016 Nov 22;7:1847. doi: 10.3389/fpsyg.2016.01847 (PMC5118620; doi:10.3389/fpsyg.2016.01847)

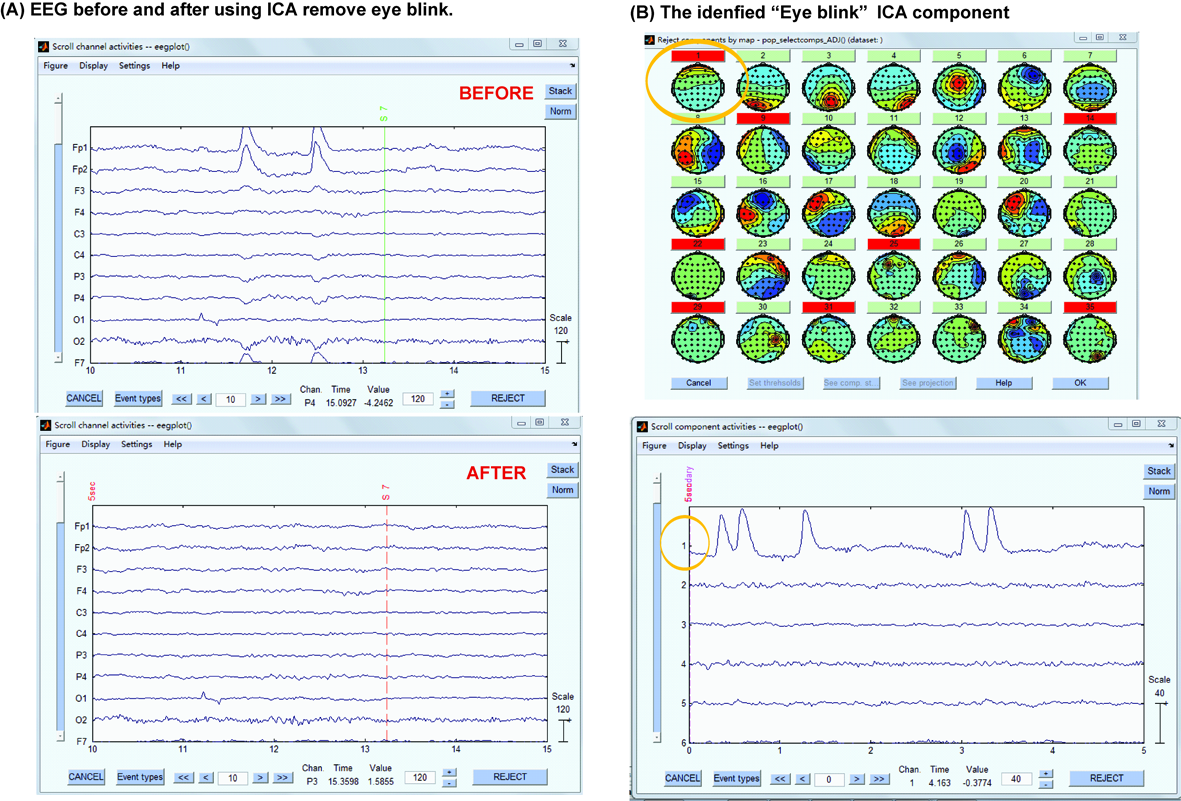

Supplement: Supplementary file 1 [file Image_1.TIF]
