# Supplementary material for: The Temporal Dynamics of Perceiving Other’s Painful Actions
Source: Front Psychol. 2016 Nov 22;7:1847. doi: 10.3389/fpsyg.2016.01847 (PMC5118620; doi:10.3389/fpsyg.2016.01847)
Supplement: Supplementary file 2 [file Table_1.DOCX]

**Supplemental Table S1.**

|  | **Dangerous Object** | **Neutral Object** |
| --- | --- | --- |
| 1 | Broken light bulb | Light bulb |
| 2 | Broken glass | Glass |
| 3 | Cactus | Tomato |
| 4 | Scorpion | Rabbit |
| 5 | Hedgehog | Chick |
| 6 | Porcupine | Plant |
| 7 | Knife | Spoon |
| 8 | Extinguished match | Lighted match |
